# Association between changes in serum alkaline phosphatase levels and radiographic progression in ankylosing spondylitis

**DOI:** 10.1038/s41598-023-36340-9

**Published:** 2023-06-05

**Authors:** Tae-Hwan Kim, Seo Young Park, Ji Hui Shin, Seunghun Lee, Kyung Bin Joo, Bon San Koo

**Affiliations:** 1 Department of Rheumatology, Hanyang University Hospital for Rheumatic Diseases, Seoul, South Korea; 2 Department of Statistics and Data Science, Korea National Open University, Seoul, South Korea; 3 Department of Radiology, Hanyang University Hospital for Rheumatic Diseases, Seoul, South Korea; 4 Division of Rheumatology, Department of Internal Medicine, Inje University Seoul Paik Hospital, Inje University College of Medicine, 9, Mareunnae-ro, Jung-gu, Seoul, 04551 South Korea

**Keywords:** Ankylosing spondylitis, Bone

## Abstract

This retrospective study evaluated the electronic medical records of patients with ankylosing spondylitis (AS) (January 2001–December 2018) to determine the relationship between serum alkaline phosphatase (ALP) levels and radiographic changes over time. Longitudinal data, including serum ALP levels, were imputed by linear interpolation at 3-month intervals. Among the serum ALP levels calculated for 8 years prior to modified Stoke Ankylosing Spondylitis Spinal Score (mSASSS) measurement, those having the highest beta coefficient with the mSASSS were selected in the correlation between ALP and longitudinal mSASSS. Linear mixed models with the selected serum ALP levels, mSASSS, and clinical variables were investigated. We included 1122 patients (mean follow-up, 8.20 [standard deviation: 2.85] years). The serum ALP level from 5 years and 3 months prior showed the highest beta coefficient with the mSASSS. In the linear mixed model, the serum ALP level at 5 years and 3 months before radiographic changes was significantly associated with the mSASSS (β = 0.021, 95% confidence interval: 0.017–0.025, *p* < 0*.*001). Serum ALP levels measured approximately 5 years before may be a surrogate marker for predicting spinal radiographic changes. Long-term prospective clinical and experimental studies of > 5 years are required for biomarker discovery or therapeutic research on AS radiographic progression.

## Introduction

Ankylosing spondylitis (AS) is chronic arthritis characterised by stiffness of the spine and the sacroiliac joints. Inflammation and new bone formation in the spine over a prolonged period of time, which eventually results in ankylosis, are important features of AS^1,2^. In terms of new bone formation, changes in the bone, ligament, and enthesis progress through complex mechanisms, including osteolysis, osteoproliferation, and osteosclerosis. These changes occur at various sites of the spine over an extended period in patients with AS and can be identified as osteitis, bone marrow oedema, or syndesmophytes^3^.

Substances related to bone metabolism, such as bone alkaline phosphatase (ALP), Dickkopf-1, sclerostin, bone morphologic protein, and receptor activator of nuclear factor kappa-Β (RANK)/receptor activator of nuclear factor kappa-Β ligand (RANKL), have been suggested as potential biomarkers for AS^4–6^. Among these, bone-specific ALP is likely a marker indicative of the net effect of bone turnover. ALPs are homodimeric enzymes that play an important role in catalytic activity and exist as four distinct isoenzymes: tissue non-specific alkaline phosphatase (TNAP), and placental, intestinal, and germ cell ALP^7^. Regarding the role of ALP, a recent study suggested that TNAP was associated with bone mineralisation and could be used as a therapeutic target for bone formation in patients with AS^8^. In addition, the study demonstrated a significant correlation between radiographic progression and bone-specific TNAP in two cohorts, suggesting that bone-specific TNAP is a potential prognostic biomarker for radiographic progression in patients with AS.

However, radiographic changes in AS are the result of a sequence of events, including inflammation, repair, and new bone formation in the spine^2,5,9^. The changes in bone metabolism may occur earlier than those that can be identified on radiographs. Although studies have evaluated the changes in inflammatory markers that precede radiographic progression^10–12^, to our best knowledge, no studies have evaluated the relationship between the timing of changes in bone metabolism and radiographic progression in patients with AS. Identifying the timing of changes in bone metabolism in AS is important for studying its pathophysiology. This is not only useful for finding bone-specific biomarkers that predict radiographic progression but also for representing a period of active bone metabolism that should be addressed in clinical and experimental studies evaluating the radiographic progression of AS.

Exploring bone-related surrogate markers in AS requires comprehensive and long-term longitudinal data on clinical variables. Electronic medical records (EMRs), which store the medical and treatment data of patients over time, may be a reasonable alternative data source for investigating the relationship between long-term ALP and spinal radiographic damage. Therefore, the present study aimed to identify the relationship between the serum ALP levels and spinal radiographic changes over time using longitudinal data from the EMRs of patients with AS.

## Methods

### Data collection

This retrospective study used EMRs obtained from a single centre. All data were collected from EMRs of 1280 patients registered from January 2001 to December 2018. These patients were diagnosed with AS according to the modified New York criteria at the time of their first visit^13^ and were followed up at a single centre since their diagnosis. Most of these patients visited the outpatient department approximately every 6 months for assessment of disease activity and underwent cervical and lumbar spine examinations at least every 2 years. Longitudinal data, such as inflammatory markers, disease activity, and radiographs, obtained during follow-ups, as well as data on baseline characteristics, such as sex, date of birth, human leucocyte antigen (HLA)-B27 positivity, diagnosis of peripheral arthritis or uveitis, and smoking status, were collected. The Hanyang University Seoul Hospital Institutional Review Board approved this study (HYUH 2018-07-007). All patient data were de-identified, and the requirement for informed consent was waived by the Institutional Review Board of Hanyang University Seoul Hospital considering the retrospective nature of this study. This study included only anonymised patient data, and the study was performed in accordance with the Declaration of Helsinki.

Among the 1280 patients, those with two or more available records of serum ALP and the modified Stoke Ankylosing Spondylitis Spinal Score (mSASSS) and an interval of > 90 days between the two records were included. Longitudinal data were imputed by linear interpolation at 3-month intervals. Laboratory data, such as the aspartate transaminase (AST), alanine transaminase (ALT), and C-reactive protein (CRP) levels, were also imputed at 3-month intervals for longitudinal analysis. The AST and ALT levels were included as clinical variables for statistical correction of liver-related diseases. In a previous study, cumulative CRP 24 months and log CRP 18 months earlier had a significant correlation with the mSASSS^10^. Therefore, cumulative CRP at 24 months and CRP at 18 months were added as clinical variables to the statistical model.

### Assessment of mSASSS on radiograph

The mSASSS was independently evaluated on cervical and lumbar spine radiographs by two radiologists (S. Lee and K. Joo) who were blinded to patient information. The mSASSS was assigned as follows: 1 point for erosion, sclerosis, and squaring; 2 points for syndesmophyte; and 3 points for ankylosis at each vertebral corner in the lateral view of the cervical and lumbar corners. As there are 24 cervical and lumbar vertebral corners, the maximum mSASSS was 72 points, indicating total ankylosis of the spine. Missing scores resulted if the vertebral corner could not be read due to compression fracture, surgery, or osteophyte. Missing scores with ≤ 3 vertebral corners were substituted by the mean score of vertebral corners in the same spinal segment^14,15^. The intra-observer reliability with consistency and inter-observer reliability with agreement between the two readers was excellent (intraclass coefficient [ICC]: 0.978, 95% confidence interval [CI] 0.976–0.979; and ICC: 0.946, 95% CI 0.941–0.950, respectively)^16–18^.

### Statistical analysis

Variables are summarised as means (standard deviations) or counts (percentages). Statistical significance was set at *p* < 0*.*05. The correlation between the serum ALP levels and longitudinal mSASSS was evaluated and the relationship between selected changes in the serum ALP levels and the mSASSS was investigated in a linear mixed model including clinical variables. The mSASSS was measured approximately every 2 years; however, the frequency varied across the patients, and even in the same patient, the frequency changed with disease progression. For longitudinal data analysis, we used the fixed, consistent person-time (person—90 days) as the unit of analysis because we did not have to assume non-informative censoring and to create a counting process and we could then apply mixed effect models that account for the clustering effect of measurements within each patient. For this purpose, at each time point of every 90 days, we used two mSASSS evaluation data that were the closest to that time point (one right before, one right after). We used the weighted average of these two mSASSS values such that the weights reflect the closeness of the mSASSS evaluation timing. This is essentially the same as linear interpolation using the two closest mSASSS values. The mSASSS value imputed in such a manner always lies between the two observed mSASSS values, which results in very plausible imputed values.

First, an initial linear mixed model was constructed with the mSASSS value as the response variable at each interval and the baseline mSASSS and time (t) as the explanatory variables: mSASSS (t) ~ baseline mSASSS + t. A random intercept was used to correct for clustering effects between observations in the same patient interval. Subsequently, the baseline serum ALP levels and serum ALP levels determined at 3, 6, …, 93, or 96 months (8 years) before the mSASSS measurements were added. The resulting statistical model was expressed as follows: mSASSS (t) ~ baseline mSASSS + t + serum ALP (t-lag t) + baseline serum ALP. Based on this model, beta coefficient estimates and 95% CIs of the serum ALP levels at 24 lagged intervals were obtained. Among these, the time interval with the highest beta coefficient with statistical significance was selected.

The time variable indicates the time since the baseline. Especially, this model’s outcome variable was the mSASSS value at time t, and this outcome was explained by the baseline mSASSS and time (t). The unit of the time variable was 90 days, i.e., t = 1 at 90 days, t = 2 at 180 days, t = 3 at 270 days, and so on. The model can be written as mSASSS (t) ~ baseline mSASSS + t (without the random intercept and error term). More specifically, our model indicates that the expected value of mSASSS at 90 days since baseline was beta_0 + beta_1*baseline mSASSS + beta_2*1. The expected value of mSASSS at 180 days since baseline was beta_0 + beta_1*baseline mSASSS + beta_2*2.

The initial model included all patients with both ALP and mSASSS measured for at least 90 days (n = 1122). All patients included in the initial model had at least six 90-day intervals (= 540 days) of data. Subsequent models that include the lagged variable only included patients with lagged ALP measurement available for at least one interval. For example, if the model included 5-year 3-month lagged ALP, it only included patients with at least 5 years and 3 months of recorded data. We considered 3, 6, … 96 months as the length of lag, and among them, measurement at 63 months (i.e., 5 years and 3 months) was selected based on the magnitude of the beta coefficient.

Second, the serum ALP at the selected delay time was entered into the following linear mixed model: mSASSS(t) ~ baseline mSASSS + t + serum ALP lag + clinical characteristics. The baseline mSASSS, time, interaction between the baseline mSASSS and time, clinical characteristics, and serum ALP levels were set as fixed effects because we believe that these variables have systematic effects on the mSASSS. Random effects were the random intercept for each patient because mSASSS measurements from the same patient tended to be similar; however, we were not focusing on each patient’s specific, systematic effect on the mSASSS. Using the random intercept for each patient, we could account for the clustering effect of mSASSS measurements from the same patient, by giving random variation to each patient similar to the sampling variation observed when randomly selecting one patient from a population. The R statistical language (version 3.6.1; R Software for Statistical Computing, Vienna, Austria) was used in the statistical analysis.

## Results

### Patients and intervals

**Figure 1:**
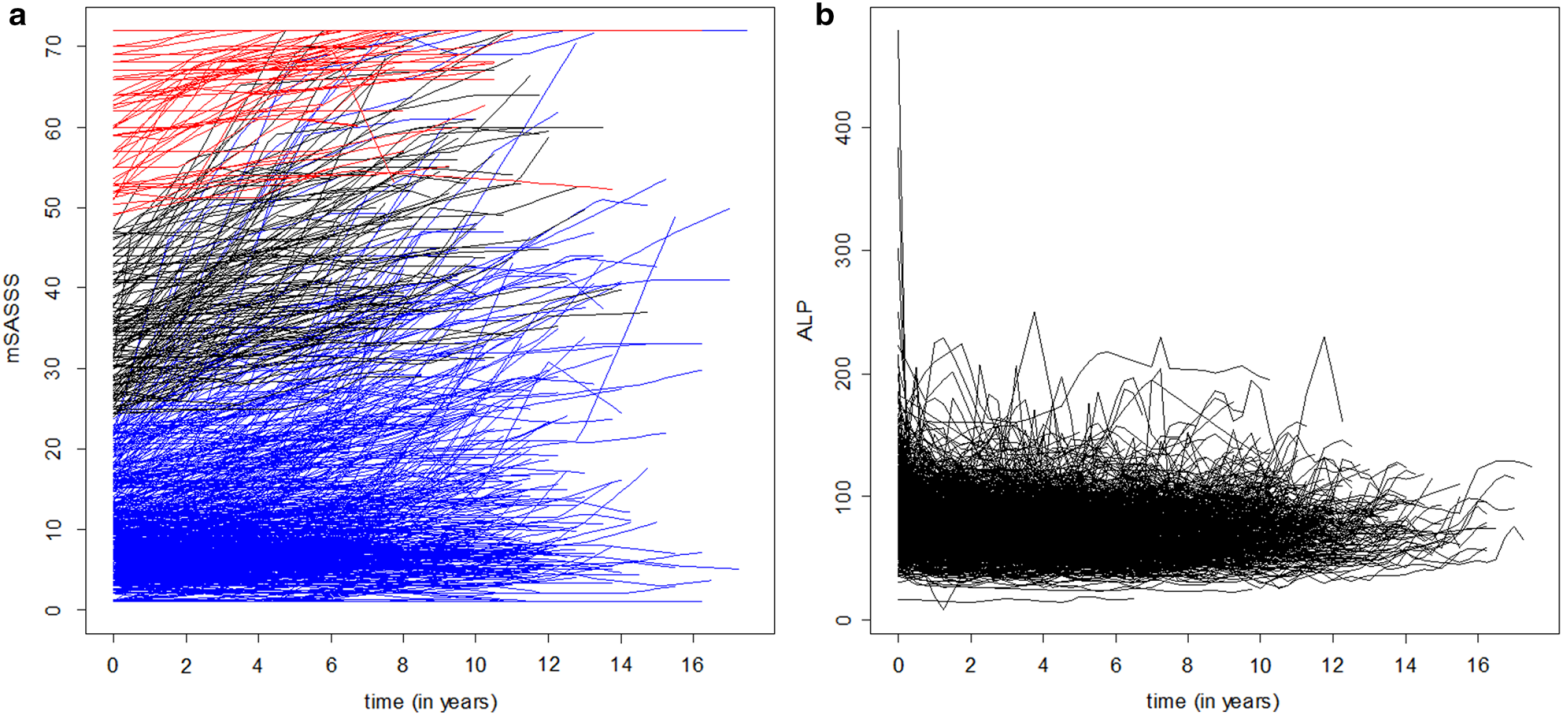
Long-term evolution of the mSASSS and ALP levels. (**a**) Changes in the mSASSS over time. The lines indicate a baseline mSASSS value of < 24 (blue lines), ≥ 24 and < 48 (black lines), and ≥ 48 (red lines) points. (**b**) ALP levels over time. ALP, alkaline phosphatase; mSASSS, modified Stoke Ankylosing Spondylitis Spinal Score

Among the 1280 patients, data from 1122 patients who met the inclusion criteria were examined. The baseline clinical characteristics of the patients are shown in Table 1. The mean follow-up period was 8.20 (standard deviation [SD]: 2.85) years. The mean number of mSASSS evaluations was 4.58 (SD: 1.21), and the average interval between mSASSS evaluations was 2.34 (SD: 0.96) years. From the longitudinal data of serum ALP in the 1122 patients, 37,852 values evaluated at 3-month intervals were obtained. Longitudinal data of the mSASSS and serum ALP levels are presented in Fig. 1. The mSASSS increased generally in most patients (Fig. 1a), although the baseline mSASSS showed high variability among patients. Serum ALP levels showed different patterns of changes in each patient (Fig. 1b). Twenty patients were randomly selected; their serum ALP levels are shown in Supplementary Fig. S1.

**Table 1:** Baseline clinical characteristics of the 1,122 included patients.

Variable	N	Value
Age at diagnosis, mean (SD), years	1122	32.00 (9.00)
Female sex, n (%)	1122	130 (11.59)
Follow-up duration, mean (SD), years	1122	8.20 (2.85)
HLA-B27 positivity, n (%)	1118	1079 (96.51)
Eye involvement, n (%)	946	361 (38.16)
Peripheral joint involvement, n (%)	936	401 (42.84)
Duration treated with TNF inhibitors per patient, mean (SD), years	627	3.14 (3.80)
ALP, mean (SD), U/L	1122	84.97 (29.94)
AST, mean (SD), U/L	1122	22.21 (16.97)
ALT, mean (SD), U/L	1122	20.81 (10.36)
CRP, mean (SD), mg/dL	1110	2.19 (2.50)
mSASSS, mean (SD)	1122	14.68 (16.33)

ALP, alkaline phosphatase; ALT, alanine aminotransferase; AST, aspartate aminotransferase; HLA, human leucocyte antigen; mSASSS, modified Stoke Ankylosing Spondylitis Spinal Score; SD, standard deviation;

TNF, tumour necrosis factor.

### Models for the correlation between mSASSS and serum ALP levels over time

An initial mixed model was constructed with mSASSS as the outcome and baseline mSASSS and time variables having values at 3-month intervals as explanatory variables (Table 2). Both explanatory variables showed a positive correlation with the mSASSS (β = 1.075, 95% CI 1.055–1.094, *p* < 0*.*001 and β = 0.208, 95% CI 0.205–0.211, *p* < 0*.*001, respectively).

**Table 2:** Initial linear mixed model for mSASSS as outcome.

Term	Beta estimate	95% confidence interval		*p*-value
		LB	UB	
(Intercept)	− 1.151	− 1.584	− 0.717	< 0.001
Baseline mSASSS	1.075	1.055	1.094	< 0.001
Time*	0.208	0.205	0.211	< 0.001

*3-month intervals.

LB, lower bound; mSASSS, modified Stoke Ankylosing Spondylitis Spinal Score; UB, upper bound.

### Significantly altered serum ALP levels were related to mSASSS

**Figure 2:**
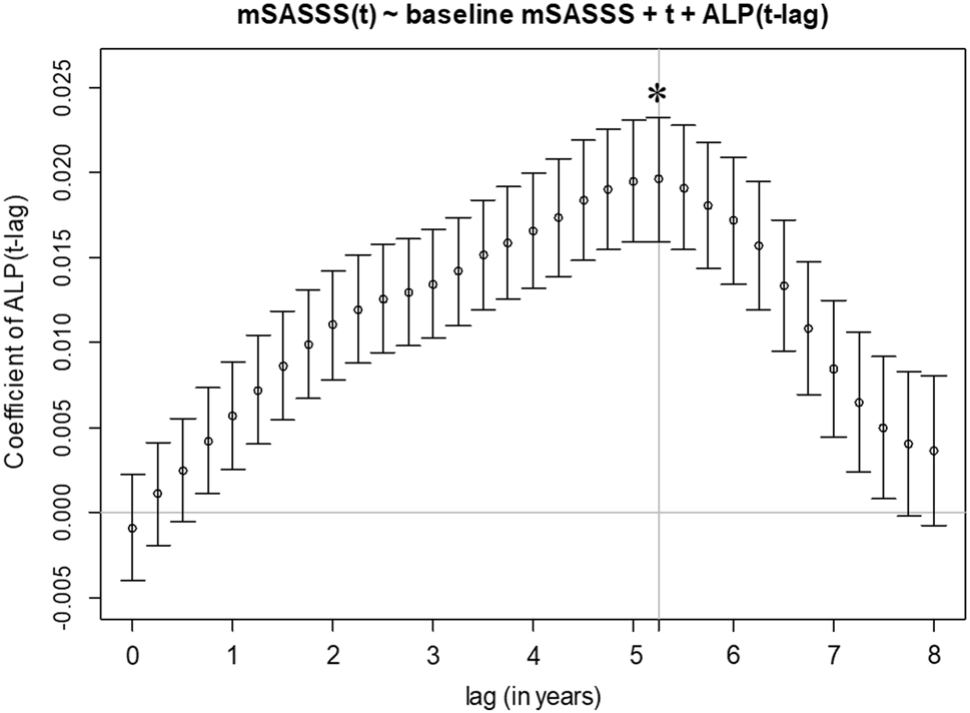
Beta coefficients of ALP levels to calculate the mSASSS. ALP levels 5 years and 3 months before the measured mSASSS had the highest significant beta coefficient (0.020, 95% confidence interval: 0.016–0.023) with the mSASSS (asterisk). ALP, alkaline phosphatase; mSASSS, modified Stoke Ankylosing Spondylitis Spinal Score.

Beta coefficients and 95% CIs over time were obtained for up to 8 years by adding the serum ALP levels imputed at 3-month intervals to the initial linear mixed model (Fig. 2 and Supplementary Table S1). A significant beta coefficient was obtained from the serum ALP levels 9 months before radiographic changes (β = 0.004, 95% CI 0.001–0.007). Among beta coefficients at 3, 6, 9…0.93 or 96 months, the highest beta coefficient was observed at 5 years and 3 months before radiographic changes (β = 0.020, 95% CI 0.016–0.023). Therefore, the serum ALP level at 5 years and 3 months prior to measuring mSASSS was selected for the final models.

### Relationship between significantly altered serum ALP level and mSASSS

A linear mixed model that included baseline characteristics was constructed using the selected 5-year and 3-month serum ALP levels before mSASSS measurement (Table 3). In Model 1, the 5-year and 3-month serum ALP levels showed a significant correlation with the mSASSS (β = 0.020, 95% CI 0.016–0.023, *p* < 0*.*001).

**Table 3:** Linear mixed models including clinical variables.

	Model 1			*p*-value	Model 2			*p*-value	Model 3			*p*-value
	Beta estimate	95% CI			Beta estimate	95% CI			Beta estimate	95% CI		
		LB	UB			LB	UB			LB	UB	
(Intercept)	− 3.198	− 3.988	− 2.407	< 0.001	− 3.644	− 7.418	0.130	0.058	− 3.759	− 7.519	0.000	0.050
Baseline mSASSS	1.095	1.064	1.126	< 0.001	1.066	1.030	1.102	< 0.001	1.066	1.030	1.102	< 0.001
Time*	0.211	0.205	0.216	< 0.001	0.216	0.210	0.221	< 0.001	0.218	0.212	0.223	< 0.001
Serum ALP 5 years and 3 months earlier	0.020	0.016	0.023	< 0.001	0.020	0.017	0.024	< 0.001	0.021	0.017	0.025	< 0.001
Cumulative CRP over 24 months					− 0.023	− 0.031	− 0.015	< 0.001				
Log CRP 18 months earlier									− 0.117	− 0.247	0.014	0.079
TNF inhibitor treatment					− 0.891	− 1.061	− 0.720	< 0.001	− 0.850	− 1.020	− 0.680	< 0.001
AST 5 years and 3 months earlier					0.001	− 0.007	0.010	0.736	0.001	− 0.007	0.010	0.794
ALT 5 years and 3 months earlier					0.000	− 0.006	0.005	0.938	0.000	− 0.006	0.005	0.907
Female sex					− 1.113	− 3.152	0.926	0.284	− 1.065	− 3.096	0.966	0.304
Eye involvement					3.008	1.791	4.225	< 0.001	2.973	1.761	4.185	< 0.001
Peripheral joint involvement					− 2.046	− 3.262	− 0.831	0.001	− 2.078	− 3.288	− 0.867	0.001
HLA-B27 positivity					− 0.625	− 4.220	2.971	0.733	− 0.558	− 4.139	3.023	0.760
Smoking (ref: non-smoker)												
Ex-smoker					3.136	1.594	4.678	< 0.001	3.139	1.603	4.675	< 0.001
Smoker					2.183	0.691	3.676	0.004	2.159	0.673	3.646	0.004

*3-month intervals.

ALP, alkaline phosphatase; ALT, alanine aminotransferase; AST, aspartate aminotransferase; CI, confidence interval; CRP, C-reactive protein; HLA, human leucocyte antigen; LB, lower bound; mSASSS, modified Stoke Ankylosing Spondylitis Spinal Score; TNF, tumour necrosis factor; UB, upper bound.

In Model 2, which included cumulative CRP for 24 months and clinical variables, the beta estimate of lagged serum ALP levels was similar to that of Model 1 (β = 0.020, 95% CI 0.017–0.024, *p* < 0*.*001). Cumulative CRP levels over 24 months and tumour necrosis factor inhibitor (TNFi) treatment negatively correlated with mSASSS (β = − 0.023, 95% CI − 0.031 to − 0.015, *p* < 0*.*001 and β = − 0.891, 95% CI − 1.061 to − 0.720, *p* < 0*.*001, respectively). Eye involvement and peripheral joint involvement showed significant positive and negative correlations with mSASSS, respectively. However, there was no significant correlation between female sex and HLA-B27 levels. Smoking was significantly positively correlated with mSASSS.

In Model 3, which included the 18-month log CRP values, the beta estimate of lagged serum ALP levels showed similar values to those in Models 1 and 2 (β = 0.021, 95% CI 0.017–0.025, *p* < 0*.*001). As in Model 2, the clinical variables in Model 3, such as TNFi treatment, eye involvement, peripheral joint involvement, and smoking, also showed significant correlations with the mSASSS. However, the 18-month log CRP value did not show any significant correlation with the mSASSS.

## Discussion

The serum ALP levels 5 years and 3 months before spinal radiographic changes were significantly correlated with radiographic progression. This is a longer period than that defined by CRP and radiographic progression, which correlated with the 2-year interval^10–12^. Although non-specific serum ALP levels were measured, these results provide evidence for the timing of active bone metabolism in patients with AS, demonstrating the need for long-term, broad clinical and experimental studies to study bone formation in the pathogenesis of AS.

Biomarkers of radiographic progression in AS are difficult to validate in prospective cohorts because they require long observation periods, and the effects of various factors, such as disease status and treatment, are difficult to control^3,8,19^. Furthermore, there are limitations in predicting when to measure bone-specific biomarkers because it is not known when bone metabolism is highly active. Several studies have reported an increase in the ALP levels in patients with AS^20,21^, but very few studies have shown an association between ALP levels and radiographic progression in patients with AS^8,22^. Recently, Liu et al.^8^ provided experimental evidence that TNAP is related to mesenchymal stem cell mineralisation in patients with AS. Further, the authors verified the relationship between the serum level of bone-specific TNAP and radiographic changes in cohorts^8^. However, as bone turnover and radiographic progression do not occur simultaneously, a more sophisticated statistical method using longitudinal data is needed to validate their study.

Here, we considered previous studies that identified a relationship between CRP levels and radiographic progression over time^10,16,17^. In one study, the correlation between inflammatory markers and radiographic progression at various time points was investigated using EMR data accumulated over an extended period^10^. Although symptoms were well controlled, spinal radiographic damage significantly correlated with increased inflammation in the 2 years before it was identified. Our approach, similar to the previous studies designs, focused on serum ALP levels. Interestingly, the beta coefficients between mSASSS and serum ALP levels were not significant within 6 months of mSASSS measurement. However, the beta coefficients increased 9 months prior and peaked at 5 years and 3 months prior. The serum ALP levels at approximately 5 years before disease progression may act as a predictive marker for spinal radiographic changes. These results provide insight into bone metabolism in relation to the pathogenesis of AS. Changes in bone metabolism may begin much earlier than radiographic damage. Furthermore, this insight may contribute to optimising the timing of treatment and measuring the effectiveness of drugs that inhibit bone turnover to prevent radiographic progression^23–25^.

TNFi treatment can slow radiographic progression by reducing inflammation^17,26^. However, when looking at the lifelong trend of mSASSS, the role of TNFi is far from complete interference with the progression of spinal damage and ankylosis. Although inflammation plays a key role in the progression of AS, it does not appear to account for most of the radiographic progression. Our results suggest that factors related to bone metabolism, such as ALP, are correlated with radiographic progression. Several laboratory studies have supported the hypothesis of inflammation-independent new bone formation in AS. The involvement of fibroblast-like synoviocytes in bone remodelling in spondyloarthritis appears to be mostly associated with inflammation-independent stromal alterations^27^. Furthermore, the expression of RANKL, osteoprotegerin, and RANK in peripheral synovitis of spondyloarthritis was independent of systemic and local inflammation^28^. This study may clinically corroborate these theories, but more experimental and clinical studies are needed on the causal relationship between inflammation and bone changes in AS.

Real-world data based on EMRs seem to be suitable for analysing data related to long-term radiographic progression in patients with AS^10,16–18,29^. However, the variables collected in retrospective studies are inevitably limited. In particular, measuring the serum ALP levels instead of bone-specific ALP levels was the best method in this exploratory study. Although variables related to liver function, such as AST and ALT, were statistically adjusted, it was difficult to suggest that the serum ALP level is a biomarker of radiographic progression in this model considering the long-term effects of various confounders.

Although there were differences in the statistical methods used here and in a previous study investigating radiographic progression in patients treated with TNFi^17^, the present study also showed that TNFi treatment correlated with the slowing of radiographic progression. Eye involvement has likewise been associated with increased radiographic progression. In our study, patients with peripheral joint involvement appeared to have less spinal damage, as observed in previous studies. Smoking significantly correlated with spinal radiographic progression in our patients with AS, as has been reported in many prior studies^11,30^. However, cumulative CRP levels over 24 months negatively correlated with radiographic progression. This may be associated with the dramatic therapeutic effect of nonsteroidal anti-inflammatory drugs or TNFi in patients with high baseline CRP levels.

The current study has some limitations. First, it should be noted that the serum ALP level identified is a net effect of activity on bone turnover and is also produced by other organs in patients with AS. Therefore, in the interpretation of these results, the characteristics of serum ALP levels deriving from different organs should be considered. Second, bone turnover may be related to osteopenia or osteoporosis as well as to spinal radiographic changes. Although most patients with AS were young and of the male sex, adjustment for bone quality may be necessary for statistical analysis. Third, drugs related to bone metabolism, such as steroids or osteoporosis medications, were not considered. Finally, the variables for obesity or metabolic diseases were not considered in the statistical model.

Serum ALP levels obtained approximately 5 years before disease progression correlated with spinal radiographic changes in retrospective longitudinal EMR data. In the future, more than 5 years of prospective clinical and experimental studies are needed to explore bone metabolism and new bone formation-related biomarkers of AS or to identify therapeutic agents for radiographic progression.

## Supplementary Information

Supplementary Information.

## Data Availability

The datasets used and/or analysed during the current study are available from the authors (Bon San Koo [koobonsan@gmail.com] and Tae-Hwan Kim [thkim@hanyang.ac.kr]) on reasonable request. Transfer of data must be approved by the Hanyang University Seoul Hospital Institutional Review Board.
