# Supplementary material for: Association between changes in serum alkaline phosphatase levels and radiographic progression in ankylosing spondylitis
Source: Sci Rep. 2023 Jun 5;13:9093. doi: 10.1038/s41598-023-36340-9 (PMC10241912; doi:10.1038/s41598-023-36340-9)
Supplement: Supplementary file 1 — Supplementary Information. [file 41598_2023_36340_MOESM1_ESM.docx]

**Supplemental Online Content**

**Figure S1**. Longitudinal ALP values of 20 randomly sampled patients


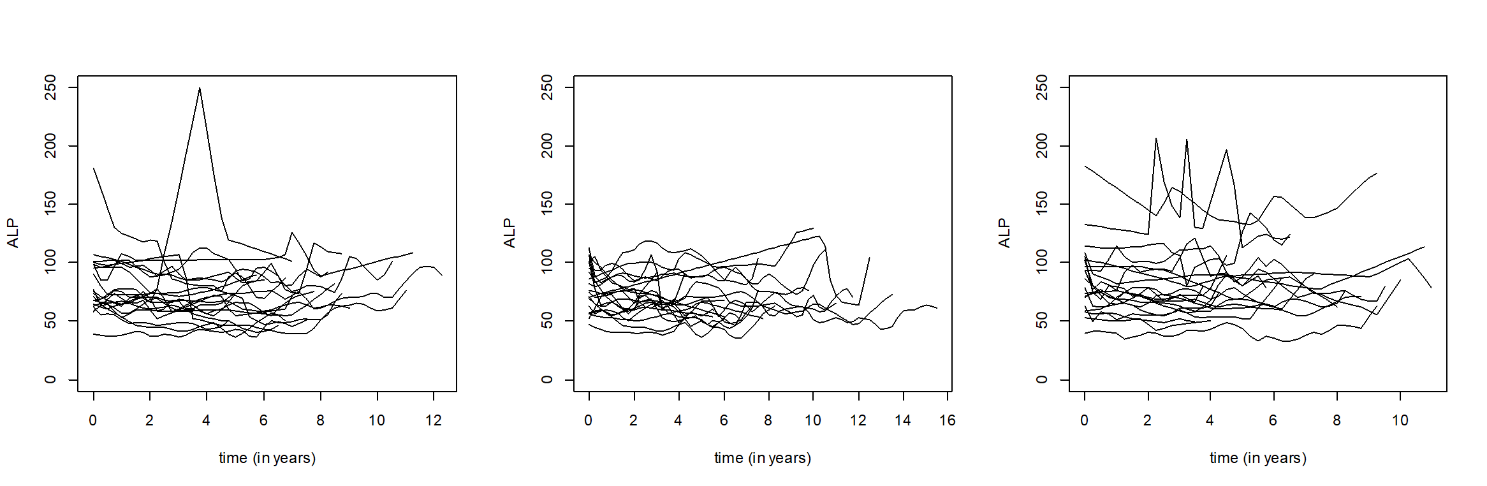


**Table S1.** Beta coefficients between mSASSS and lagged serum ALP levels.

| lag (in months) | lag (in years) | beta est. | beta 95% CI | |
| --- | --- | --- | --- | --- |
|  |  |  | LB | UB |
| 0 | 0 | -0.001 | -0.004 | 0.002 |
| 3 | 0.25 | 0.001 | -0.002 | 0.004 |
| 6 | 0.5 | 0.002 | -0.001 | 0.006 |
| 9 | 0.75 | 0.004 | 0.001 | 0.007 |
| 12 | 1 | 0.006 | 0.003 | 0.009 |
| 15 | 1.25 | 0.007 | 0.004 | 0.010 |
| 18 | 1.5 | 0.009 | 0.005 | 0.012 |
| 21 | 1.75 | 0.010 | 0.007 | 0.013 |
| 24 | 2 | 0.011 | 0.008 | 0.014 |
| 27 | 2.25 | 0.012 | 0.009 | 0.015 |
| 30 | 2.5 | 0.013 | 0.009 | 0.016 |
| 33 | 2.75 | 0.013 | 0.010 | 0.016 |
| 36 | 3 | 0.013 | 0.010 | 0.017 |
| 39 | 3.25 | 0.014 | 0.011 | 0.017 |
| 42 | 3.5 | 0.015 | 0.012 | 0.018 |
| 45 | 3.75 | 0.016 | 0.013 | 0.019 |
| 48 | 4 | 0.017 | 0.013 | 0.020 |
| 51 | 4.25 | 0.017 | 0.014 | 0.021 |
| 54 | 4.5 | 0.018 | 0.015 | 0.022 |
| 57 | 4.75 | 0.019 | 0.015 | 0.023 |
| 60 | 5 | 0.019 | 0.016 | 0.023 |
| 63 | 5.25 | 0.020 | 0.016 | 0.023 |
| 66 | 5.5 | 0.019 | 0.015 | 0.023 |
| 69 | 5.75 | 0.018 | 0.014 | 0.022 |
| 72 | 6 | 0.017 | 0.013 | 0.021 |
| 75 | 6.25 | 0.016 | 0.012 | 0.019 |
| 78 | 6.5 | 0.013 | 0.009 | 0.017 |
| 81 | 6.75 | 0.011 | 0.007 | 0.015 |
| 84 | 7 | 0.008 | 0.004 | 0.012 |
| 87 | 7.25 | 0.006 | 0.002 | 0.011 |
| 90 | 7.5 | 0.005 | 0.001 | 0.009 |
| 93 | 7.75 | 0.004 | 0.000 | 0.008 |
| 96 | 8 | 0.004 | -0.001 | 0.008 |
